# Strengthening the Delivery of Physical Healthcare for Adults Living With Serious Mental Illness – A Qualitative Description of Patient and Family Member Perspectives

**DOI:** 10.1111/hex.70224

**Published:** 2025-03-25

**Authors:** Munazzah Ambreen, Christopher Canning, Brian Lo, Sri Mahavir Agarwal, David Castle, Barna Konkolÿ‐Thege, Frank Sirotich, Sanjeev Sockalingam, Tania Tajirian, Philip G. Tibbo, Mary Rose van Kesteren, Caroline Walker, Vicky Stergiopoulos

**Affiliations:** ^1^ Centre for Addiction and Mental Health Toronto Ontario Canada; ^2^ Waypoint Research Institute, Waypoint Centre for Mental Health Care, Penetanguishene Ontario Canada; ^3^ Institute of Health Policy, Management and Evaluation University of Toronto Toronto Ontario Canada; ^4^ Department of Psychiatry University of Toronto Toronto Ontario Canada; ^5^ School of Medicine University of Tasmania Hobart Australia; ^6^ Tasmanian Centre for Mental Health Service Innovation, Tasmanian Health Service Hobart Australia; ^7^ Canadian Mental Health Association Toronto Branch Toronto Ontario Canada; ^8^ Factor‐Inwentash Faculty of Social Work University of Toronto Toronto Ontario Canada; ^9^ Department of Family and Community Medicine University of Toronto Toronto Ontario Canada; ^10^ Department of Psychology and Neuroscience Dalhousie University Halifax Nova Scotia Canada; ^11^ Department of Psychiatry Dalhousie University Halifax Nova Scotia Canada

**Keywords:** integrated care, qualitative design, reverse integration, serious mental illness, service user perspective

## Abstract

**Background:**

Individuals with serious mental illness (SMI) have higher rates of comorbid physical health conditions, poorer associated health outcomes, and die on average 10–20 years earlier than the general population. This qualitative study aimed to explore the perspectives and experiences of adults living with SMI and family members with accessing physical healthcare within primary and mental health settings in Canada.

**Methods:**

We conducted a qualitative descriptive study using semi‐structured interviews with 20 adults living with SMI and five focus groups with 18 family members between July 2023 and April 2024. After coding by two authors, thematic analysis was completed with the support of a data analysis team to identify overarching themes capturing participant experiences with accessing physical healthcare, care needs and preferences.

**Results:**

Four main themes emerged from participant narratives: (1) The centrality of mental health problems in the lives of people with SMI; (2) Challenges in accessing physical healthcare; (3) The role of families in supporting access to care; (4) Perceived health priorities and preferences. There was a high degree of congruence between the perspectives of individuals living with SMI and family members. Both participant groups described challenges accessing primary care settings, fragmented health services, and a desire for person‐centred, whole‐person health within mental health settings, with family member support where available.

**Conclusions:**

Findings from this study highlight the need for advancing the integration of physical healthcare within mental health settings for adults living with SMI, who are less likely to engage with community‐based primary care services. Enhanced access to physical healthcare could leverage multidisciplinary resources in these settings and partnerships with families. These findings can inform efforts to provide whole‐person healthcare for individuals experiencing SMI.

**Patient or Public Contribution:**

The study team collaborated closely with community organizations and individuals with lived experience at every stage of this research. This included contributions to the funding proposal, the study protocol, participant recruitment, study materials, data analysis and preparing the manuscript. Individuals with lived experience and family members actively participated in management and project meetings for the duration of the study.

## 1 Introduction

The Mental Health Commission of Canada estimates that approximately 5% of Canadians experience serious mental illness (SMI) at any given time [1, 2]. Individuals living with SMI have higher rates of chronic physical health conditions, physical multi‐morbidity, greater acute care use, poorer health outcomes, and, on average, a lifespan 10–20 years shorter than the general population [3, 4, 5, 6, 7, 8, 9, 10, 11, 12, 13]. In Canada, as in many other countries, these challenges are exacerbated by service fragmentation and poor health service engagement, which hinder timely screening and appropriate management of chronic health conditions in this population [14, 15, 16]. Consequently, individuals living with SMI experience poor quality of care and growing health disparities, compounded by poverty, housing instability, criminalization and pervasive stigma [17, 18, 19, 20, 21].

Over the past 40 years, various models of collaborative physical and mental healthcare have been implemented internationally to improve access to mental healthcare for individuals with mild and moderate mental health conditions, by integrating mental health professionals in primary care settings [22, 23, 24, 25]. Less is known about how best to integrate physical and mental healthcare delivery for individuals experiencing SMI, typically served by specialty mental health services, such as long‐stay psychiatric hospital units and assertive community treatment teams [9, 24, 25]. Recent literature has described such ‘reverse integration’ models, which typically embed primary care providers within mental health settings, along with support for patient self‐management and care coordination [26, 27, 28, 29, 30]. These models have been shown to have positive effects on the quality of care, preventive care and chronic disease management, and mixed but promising findings on the physical functioning of adults living with SMI [26, 27, 28, 29, 31, 32, 33]. Furthermore, to address physical health comorbidities, multi‐morbidity, and the mortality gap in this population, several countries have advanced policies or initiatives integrating physical and mental healthcare delivery within mental health service settings for this population [30, 31, 34, 35, 36]. Yet despite increasing recognition of the access barriers to physical healthcare and the impact of living with multiple comorbid conditions among adults experiencing SMI, much remains to be done to address the mortality gap and advance access to appropriate physical healthcare for this population, grounded in their needs and preferences [16, 37].

In Canada, within a system of universal health insurance, adults living with SMI are typically engaged with specialty mental health services, with primary care providers having a marginal role in mental health service provision in most settings. Primary and specialty mental health services are fragmented and often uncoordinated, with access barriers common in both services. To address these challenges, with no specific policy and limited practice mandates, specialized mental health services increasingly offer metabolic monitoring to patients on atypical antipsychotic medications, along with interventions to mitigate side effects related to weight gain and metabolic imbalances [35, 36]. Furthermore, large mental health service provider organizations have intensified efforts to provide integrated physical and mental healthcare in recent years, by integrating family physicians or nurse practitioners into the mental healthcare environment [35]. Yet significant knowledge and practice gaps remain, including scant literature on the perspectives and experiences of individuals living with SMI and family members with accessing physical healthcare in primary and specialty psychiatric hospitals and community mental health settings [38].

Qualitative research is well suited to answer nuanced questions such as experiences of care as well as care needs and preferences [39]. To address knowledge gaps and inform service redesign in the Canadian context, including reversed integration initiatives for adults living with SMI, this study sought to elicit patient and family experiences with accessing physical healthcare and to gain their perspectives on how best to support the physical health needs of this population within mental health settings in Canada.

## 2 Materials and Methods

Leveraging participatory approaches and a constructivism paradigm [40], we used qualitative descriptive methodology, which is often used in healthcare studies to identify participants' direct care experiences and recommendations for how a particular program or service might be improved [39, 41]. Research Ethics Board (REB) approval was obtained from CAMH (2023/057), the University of Toronto (00045386), Ontario Shores Centre for Mental Health Sciences (JREB# 23‐022‐R) and Dalhousie University (REB# 1030041). All participants provided written informed consent.

### 2.1 Participants and Recruitment

A Lived Experience Advisory Council (LEAC) was involved in all aspects of the study, and advised on the development of interview guides and study recruitment, while a data analysis subgroup, inclusive of individuals living with SMI and family members, supported data analysis and interpretation. We used purposive sampling to recruit 20 adults living with SMI and 18 family members during the study period (July 2023 to April 2024). Individuals with SMI were eligible for study inclusion if they experienced a serious mental illness (i.e., a mental health condition such as bipolar disorder or schizophrenia that ‘significantly limits a person's ability to function in their daily life’) [42]; were able to speak English and provide informed consent; and were 18 years or older. As we sought to engage individuals with severe disabilities, we recruited participants from psychiatric hospital inpatient units, Assertive Community Treatment (ACT) teams and a First Episode of Psychosis program. Inclusion criteria for family members included age over 18, being a family member of someone diagnosed with SMI, capacity to provide informed consent and fluency in English.

Potential participants were contacted through ACT teams, community mental health organizations and psychiatric hospitals in Ontario and Nova Scotia. Recruitment materials and the study flyer, with instructions to contact the research team if interested in participating, were disseminated widely. Eligible participants with SMI were often introduced to the study by someone in their circle of care (e.g., physician, nurse, social worker). Family member participants were recruited from community organizations and through the networks of LEAC members. A research coordinator responded to potential participant inquiries and organized an interview time following receipt of written informed consent.

### 2.2 Data Collection

We conducted in‐person individual interviews with study participants living with SMI and utilized videoconferencing for focus groups with family members using semi‐structured interview guides that were developed with input from LEAC. We conducted in‐person individual interviews with patient participants to promote a sense of safety and gain a deep understanding of individual patient experiences. We opted for focus groups with family members for feasibility purposes, and to encourage dialogue, allowing for a diversity of ideas and perspectives. The interview guide for participants living with SMI was pilot‐tested with the first four interviews and minor adjustments were made to improve interview flow. Both interview guides explored the experiences of individuals with SMI with accessing physical healthcare and perspectives on how best to integrate physical healthcare within mental health settings (See Supplementary Appendix‐1). All 20 individual interviews and five focus groups were conducted by two female research coordinators (MA, RC) who have health professional backgrounds (medicine, occupational therapy) and experience in qualitative interviewing. Interviews (lasting 40–60 min) and focus groups (lasting 60–90 min) were digitally recorded, transcribed verbatim and identified with a unique code to maintain participant confidentiality. Data collection stopped at the point of conceptual saturation [39, 43].

### 2.3 Data Analysis

Data were analysed using inductive thematic analysis [39]. Transcripts were coded to capture challenges in accessing appropriate physical healthcare, and perceived health priorities and preferences. The analysis followed six steps: (1) familiarization with data, (2) generation of initial codes, (3) searches for themes, (4) review of themes, (5) defining and naming themes and (6) report production.

Coding was completed after multiple immersive readings by two researchers; three transcripts were independently coded by team members to align on coding strategy, ensure consistency and resolve discrepancies before coding the remaining transcripts. We sought consensus in coding through discussion at biweekly data analysis team meetings over a 3‐month period. Peer debriefing with the larger research team expanded perspectives regarding our analyses, including key differences within and across participant groups. NVivo 12 software was used to apply codes across the data set. The Consolidated Criteria for Reporting Qualitative Studies (COREQ) checklist was used to guide reporting [44].

## 3 Results

Participant demographics and clinical characteristics are presented in Table 1. Participants with SMI experienced a range of physical health conditions, although it was not a criterion for recruitment. Family member participants included parents (*n* = 10), spouses (*n* = 4), siblings (*n* = 3) and children (*n* = 1). We identified four key themes and high levels of convergence within and across participant group data, reflecting shared experiences. Themes focused on the centrality of mental health problems in the lives of individuals living with SMI, their experiences of accessing physical healthcare in both primary and specialty mental health settings, the role of families in supporting access to care, and perceived care priorities and preferences. These themes and associated subthemes are further described below, supported by representative quotes.

**Table 1:** Participant demographic and clinical characteristics.

Characteristic		Participants with SMI (*N* = 20), *n* (%)	Family members (*N* = 18), *n* (%)
Age	18–30	5 (25.0)	n/a
	31–49	8 (40.0)	3 (16.7)
	50–64	5 (25.0)	5 (27.8)
	65+	2 (10.0)	10 (55.5)
Gender	Man	8 (40.0)	6 (33.3)
	Woman	8 (40.0)	12 (66.7)
	Transgender	1 (5.0)	n/a
	Gender Fluid	1 (5.0)	n/a
	Nonbinary	2 (10.0)	n/a
Sex	Female	10 (50.0)	12 (66.7)
	Male	10 (50.0)	6 (33.3)
Ethnicity	White‐European	3 (15.0)	3 (16.7)
	White‐North American	9 (45.0)	15 (83.3)
	South Asian	2 (10.0)	n/a
	South‐East Asian	3 (15.0)	n/a
	Black Caribbean	1 (5.0)	n/a
	Black African	2 (10.0)	n/a
Province	Ontario	15 (75.0)	10 (55.6)
	Alberta	n/a	3 (16.7)
	Nova Scotia	5 (25.0)	5 (27.7)
Mental illness	Schizophrenia or Schizoaffective	12 (60.0)	9
	Bipolar and related disorders, or related symptoms	6 (30.0)	8
	Obsessive‐compulsive and related disorders, or related symptoms	1 (5.0)	n/a
	Trauma‐ and stressor‐related disorders	1 (5.0)	n/a
	Major Depressive Disorder		1
Physical health diagnosis	Diabetes	4 (20.0)	2 (20.0)^a^
	Thyroid problem	1 (5.0)	1 (10.0)
	Pre‐diabetic	3 (15.0)	n/a
	Chronic pain	1 (5.0)	n/a
	Hypertension	2 (10.0)	n/a
	Anaemia	1 (5.0)	n/a
	High liver enzymes	1 (5.0)	n/a
	Asthma	2 (10.0)	n/a
	Eczema	1 (5.0)	n/a
	Obesity	1 (5.0)	1 (10.0)
	Celiac Disease	1 (5.0)	n/a
	HIV	1 (5.0)	n/a
	Kidney Disease		1 (10.0)
	Epilepsy		2 (20.0)
	Cancer		1 (10.0)
	Inflammatory disease		2 (20.0)

^a^ Ten of 18 family member participants provided information on physical health comorbidities of family members with SMI.

### 3.1 The Centrality of Mental Health Problems in the Lives of People Living with SMI

Participants living with SMI described that the impact of mental illness on their lives was overwhelming, interfering with access to both physical and mental healthcare. Participants explained that engaging with health services requires certain capabilities, including illness awareness, motivation and organizational and planning skills, and these were often compromised in this population. As this participant shared:

However, it takes me being really at the top of my game to access the things I need, and unfortunately, when I needed things the most, I was not at the top of my game, and obviously—I needed help accessing anything.

(P‐15)

The presence and impact of SMI were described as all‐encompassing by several participants, eliciting feelings of inadequacy in asking for help, and making it difficult to make and keep appointments or adhere to treatment regimens. Participants further highlighted the difficulties in navigating fragmented health services, with narrow professional scopes of practice and poor communication and care coordination between primary care and mental healthcare providers. As this participant living with SMI expressed*:*

I found in the past struggling to make it to my monthly appointment with a psychiatrist and then having a [physical] health issue as well and the [physical] health issue not being addressed [in the mental health setting].

(P‐7)

These challenges were echoed in family member narratives; as this participant described

She has been in and out of treatment centers. However, it has been exhausting. Always have to advocate because if we do not, there is a tendency to see her‐‐she is an attractive, well‐spoken young woman, and they are like, well, “she presents well.” But she cannot get herself to appointments. She can't—you know, her executive functioning is very poor. But there's a tendency to focus on what people look like and how they speak, as opposed to whether they can perform the activities of daily living.

(F‐7)

Other family members expressed that within a system that has failed to deliver integrated physical and mental healthcare, one often chose or prioritized mental health, to the detriment of physical healthcare. As one family member said

Physical care is not a main focus because, usually, it's a very distressing situation where we have to take care of the underlying mental issue here before we can even focus on anything physical.

(F‐9)

### 3.2 Challenges in Accessing Physical Healthcare

Participants living with SMI and family members described difficulty accessing physical healthcare and care coordination between mental health and primary care providers, along with variable experiences of integrated physical healthcare within mental health settings.

#### 3.2.1 Difficulty Accessing Primary Care in the Community

Despite a universal health insurance system, study participants invariably reported difficulty accessing primary care and described that common primary care practices are not suited to their needs. They also highlighted occasions when stigma associated with mental illness overshadowed the care they received in primary care settings.

The difficulty accessing primary care was commented on by several participants living with SMI, as this participant described, ‘I recently just got set up with a family physician 3 months ago, when we've been waiting for a family doctor for over a year and a half’ (P‐7). Similarly, participants commented that very brief appointments and long wait times for appointments are barriers to engagement in primary care settings. As one family member participant said, ‘What I've noticed with the care that she [daughter] receives is that it's usually only in emergency situations. So, there's very little preventative healthcare within her primary care setting’ (F‐4). Another family member participant commented:

We have a GP, but one of the things that has been a real challenge is that they've changed the system so that family doctors are not doing annual physical check appointments anymore. So, for my daughter, as a person with bipolar disorder, it's kind of hard to pay attention… So, she is kind of falling through the cracks.

(F‐15)

Participants living with SMI further noted stigma to be a significant barrier to accessing appropriate care in primary care settings. They described facing stigma and discrimination from primary care providers in the community, which discouraged them from seeking treatment. As this participant related:

I would definitely say that the stigma around people with serious mental illness is almost like you're always met with judgment or questions. When you try to explain a more serious health or mental health issue, you're met with disbelief, and that really turns a lot of people away, especially adults, from the care that they actually need…. It's one of the biggest barriers that we have to face to get treatment of any kind.

(P‐16)

Family members similarly described community‐based family physicians questioning the trustworthiness of patients with SMI. As one family member said, ‘The doctor [family physician] said to me, “You can't trust him.” And I'm thinking, “You can't do anything for him. And I can't trust him? I was sort of disgusted…”*’* (F‐9).

#### 3.2.2 Variable Access to Physical Healthcare Within Mental Health Settings

Participants living with SMI and family members reported a wide range of experiences in accessing physical healthcare within mental health settings. They noted significant gaps in communication and coordination between primary care and specialty mental healthcare providers and described that their physical health issues, when brought up in outpatient mental healthcare encounters, were often dismissed. As this participant described, ‘The psychiatrist generally just says, you know, it's [any physical health symptom] not a psychiatric issue’ (P‐6).

Conversely, physical healthcare was more accessible within long‐term inpatient psychiatric units and through some Assertive Community Treatment (ACT) teams and was highly valued, when available. One participant remarked, ‘If I needed something [support with physical health needs] like that, I would have to call my nurse practitioner at [mental health setting]. That's the only person dealing with me’ (P‐8). Similarly, as one family member described, ‘He [psychiatrist] saw her as a whole person—her epilepsy, depression, and addiction. He tried to develop a plan for her and made an effort to communicate with the other [specialists] doctors. He was the first doctor who did that’ (F‐7).

The differential access to physical healthcare between inpatient and outpatient mental health settings was remarked by several participants, with some family members expressing frustration with the loss of care continuity after discharge from psychiatric hospitals. One family member noted, ‘I remember communicating with nephrologists about renal damage from antipsychotic medication [while an inpatient], but nothing happened in the end. The issue fell through during transitions [to outpatient psychiatric care]’ (F‐12).

Other participants commented on the lack of communication and coordination between primary care and specialty mental health providers, necessitating efforts on their part to facilitate connections between providers. As one participant said, ‘There was no communication, even when I was in psychosis. I had to set up a meeting with my psychiatrist and family doctor myself. That's the only way my clinicians will get connected’ (P‐3). A family member added, ‘There's very little cross‐discussion between doctors. Each doctor tells me they are not responsible for reviewing all the medications she's on. This leaves me, an unqualified person, to manage her medication, which has been a significant problem’ (F‐2).

Finally, participants commented on the central role of nursing in identifying and supporting the physical health needs of individuals living with SMI, in both psychiatric inpatient and ACT team settings, underlining their role as approachable and trusted figures in healthcare. As one participant described, ‘My physical treatment regimen isn't necessarily only provided by doctors. But I see my treatment regimens are provided by nurses mainly’ (P‐14).

### 3.3 The Role of Families in Accessing Healthcare: Families as Care Partners

Both participants living with SMI and family members described several important roles that families assume in supporting people with SMI. They described families offering both instrumental support, such as financial support and housing, as well as health system navigation and advocacy. Family members further commented that their roles and contributions are not often recognized or leveraged, leading to missed opportunities to coordinate care for people living with SMI.

This participant expressed their dependence on family to have basic needs met and feeling trapped and hopeless without access to appropriate resources. As they described, ‘I'm stuck. There's no [family] doctor. There's no housing for me. There's nothing. You know what, if I—if my mom dies tomorrow morning, god—knock on wood, I'm done. I'm done’ (P‐6).

Family member participants similarly highlighted their key role in health and social care navigation and advocacy for individuals with SMI. These family members described coordinating care between healthcare providers to ensure that their loved ones receive the care needed. As this family member participant described, ‘I am her navigator in the health care system. I'm the advocate. I'm the organizer. I don't want to be’ (F‐17).

Another family member participant commented on their role in collecting and sharing medical information to support care coordination: ‘I have to sort of pull all the information from all the other specialists, and I go to a meeting very prepared. And so it is crazy that I feel like my sister's health is primarily on me when I'm not a medical professional’ (F‐10).

Family member participants described that dealing with the shortcomings of the health and social care systems carries a large emotional and financial toll, and the burden being at times overwhelming, often likened to a second job. Given the emotional, instrumental support and advocacy family members provide to individuals living with SMI, they described their role as care partners, although this role was not consistently recognized or valued. This participant described a positive experience of inclusion:

[The healthcare team] listened to her [daughter], and they understood that she felt we were a really important part of her team. And so they included us in just about everything that, you know, we wanted to be included in, because she said, “Yes, I would like my parents there. Yes, I need their help”. And so they very much treated us as part of the team. And that was a really nice change.

(F‐6)

Other family member participants, on the other hand, expressed that healthcare providers often overlook or dismiss their involvement. This lack of recognition can be frustrating to the care process, as family members are often key sources of support and information.

We, as family members, can offer valuable input about whether a medication is working or provide suggestions based on our observations. When you live with or care for someone, you have crucial information that is highly valuable—and even essential—for medical professionals. However, the system often prevents this information from being shared.

(F‐9)

### 3.4 Perceived Care Priorities and Preferences

In discussing the physical health needs and preferences of individuals living with SMI, both participant groups identified several areas for improvement within mental health settings, including the availability of ‘whole‐person’ health and support with chronic disease self‐management and system navigation.

#### 3.4.1 The Need for ‘Whole‐Person’ Health Within Mental Health Settings

Participants favoured integrated physical and mental health service delivery and suggested that care for individuals living with SMI within mental health settings should extend beyond traditional mental health symptom management to include preventive healthcare, chronic disease management, as well as broader aspects of health, such as exercise, diet and sexual health, tailored to the sociocultural needs and preferences of service users. Access to a variety of therapeutic and support services, including physical fitness, dietary counselling and general health advice, were considered highly beneficial in advancing whole‐person health and consequently highly desirable within mental health settings, as this participant outlined

I also feel like, I don't know if it's just because [of] my personal case with the clinic or not, but I think there's other areas of your life that we could be checking in on as well, that can open up doorways to helping people. Like even sexual health as well. I feel like that kind of gets overlooked.

(P‐8)

Further, as this participant expressed

And of course, family, psychosocial, cultural, and ethnic factors can also play into a physician's attitudes and perceptions of how to deal with treatment.

(P‐16)

A family member participant echoed the need to go beyond mental health symptom management to offer a range of physical health and health‐promoting services within mental health settings:

I think access to certain services, outpatient services, like physical fitness, exercise, things like that would be really beneficial. We have exercise and fitness programs, you know, that are catering to certain groups.

(F‐12)

#### 3.4.2 Support With Self‐Management and Service Navigation Within Mental Health Settings

In addition to accessing physical healthcare and health‐promoting services in mental health settings, participants living with SMI emphasized the importance of feeling empowered to identify and manage their physical health needs. They expressed that helping individuals living with SMI understand how to make appointments and providing guidance on navigating healthcare tasks can foster independence and self‐efficacy, while also encouraging them to seek timely help when needed. As this participant expressed,

When I go to get my blood test, all I need to do is go online and make an appointment myself. [My nurse] and [Psychiatrist] has shown me how to do that. It's pretty easy.

(P‐17)

Another participant commented on being encouraged to seek help for any health concerns they may experience,

For any physical health issues [while an inpatient], I would ask the nurse to make an appointment with the hospitalists.

(P‐10)

Participants living with SMI further spoke of the need for support navigating other specialist health services, and the importance of having access to up‐to‐date information on available services and supports. As this participant voiced:

We are in the dark about supports available to us. It's kind of just ‘Oh, your symptoms are fine. Your mental health is okay. You're good’ [implying that clinicians talk about the bare minimum]. But if you want to take more proactive steps to helping yourself and … accessing resources that are available to you [you need information about available services].

(P‐17)

Similarly, family member participants expressed the need for better health supports for people living with SMI, including more consistent case management and service navigation. As this family member participant explained

We have to pay privately for someone to come in and make those connections and be the advocate. But that was one thing that was really needed, [it] is someone who knew this system, someone [who] knew how to advocate, someone who is there by my mom's side when we couldn't be but again, we had to pay out of pocket for that.

(F‐4)

## 4 Discussion

With increased awareness of the high prevalence of physical comorbidities and multi‐morbidity among adults living with SMI, and the associated impact on quality of life, mortality and societal and personal costs, several reports have called for greater integration between physical and mental healthcare [16, 28, 29, 37]. Yet although interest in improving access to timely and appropriate physical healthcare for this population is growing internationally, the literature on the perspectives and experiences of affected individuals and family members is scant [3, 45, 46, 47]. This qualitative study explored the experiences of adults living with SMI and family members with accessing physical healthcare, and their perspectives on how best to support the physical health needs of this population within mental health settings in Canada. Our study therefore offers unique insights into the needs and preferences of affected individuals and family members in the Canadian context.

First, participants living with SMI and family members described the centrality of mental health problems in the lives of individuals living with SMI, which compromise proactive health‐seeking and health service engagement, and lead many to in this population ‘falling through the cracks’. This supports and is supported by prior international research that highlights the pervasive nature of serious mental illness and the burden of managing physical health issues while dealing with serious mental health concerns [3, 7, 45, 46, 47, 48].

Further, despite a system of universal health insurance, both patient and family member participants uniformly described difficulties with accessing timely and appropriate community‐based primary care. Common barriers to accessing physical healthcare in primary care settings in Canada include long wait times for services, and common primary care practices not suited to the needs of individuals living with SMI. Stigma from primary healthcare providers was also frequently reported, with both participant groups describing feeling judged or disbelieved, which further discouraged seeking necessary treatment in primary care settings. These challenges echo those identified in previous studies addressing the physical health needs of this population internationally, and support the rationale to integrate physical healthcare delivery within mental health settings for adults with SMI [2, 3, 4, 5, 7, 16, 21, 23, 38, 45, 47, 48].

There was a high level of congruence between participant group narratives in expressing frustration with primary care access barriers and service fragmentation in their local context. Participants living with SMI struggled to access and navigate diverse services, reporting poor communication and coordination between providers, while family members described similar challenges in coordinating care as they advocated for loved ones. Service fragmentation often led to the prioritization of mental health at the expense of physical health needs, leaving significant care needs unaddressed. Finally, participants described unmet needs for support and the central role of families in addressing basic needs, and providing service navigation and advocacy. The need for social support, in addition to professional care, has been previously identified as a key facilitator for managing health in this population in other studies [45, 48].

Some differences in perspectives and experiences also emerged. Patient participants uniformly described that their physical health needs were addressed in long‐term psychiatric inpatient units, while their experiences accessing physical healthcare within community mental health settings were more variable. Family member participants expressed concerns about their lack of inclusion in care, a concern that was not prominent in patient participant narratives.

Finally, participants emphasized the need for mental health settings to offer ‘whole‐person health’, tailored to the needs of this population. This was thought to involve, in addition to preventive health services and chronic disease management, physical fitness, sexual health and dietary counselling. They further called for support with self‐management and service navigation, and highlighted the central role of nursing in addressing their physical health needs within mental health services. A recently proposed integrative collaborative care model for adults with mental illness and physical comorbidities, set within primary care, similarly went beyond the integration of traditional physical and mental healthcare to include lifestyle interventions, adjunctive neutraceutical treatment and mind–body therapies, in keeping with the desire for whole‐person health among our participants [21].

Overall, our findings support research from other countries reporting patient preference for integrated care models and collocated services to address system fragmentation, offering insights relevant to the Canadian context [47]. As calls for integrated physical and mental healthcare models multiply, it will be important to recognize that integration is needed at various levels, including government agencies, healthcare organizations, clinics and direct service provision to patients. It is also important to highlight the different types and level of integration, from off–site collaboration and care coordination to colocation of services, and finally fully integrated physical and mental healthcare, where teams of providers develop collaborative care plans [37, 49, 50].

Integrating physical healthcare within mental health services is not without challenges. Implementation challenges in prior research include securing financial resources, maintaining effective use of clinical information systems, staff turnover and balancing immediate care needs with population‐based management and preventive care [31, 51, 52, 53, 54]. Despite these challenges, working with patients, family members and providers in service redesign is essential. In these efforts, it will be important to consider the complexity of care, including access to disease‐specific, specialist delivered care, and the interaction of different health professionals and subspecialists across different levels and types of care [13, 37, 49]. Last but not least, it is important that emerging models recognize that the nature and quality of relationships in healthcare influence the process and outcomes of care. Relationship‐centred care (RCC) is a valuable conceptual framework to consider when addressing comorbid mental and physical health problems with patients or within healthcare organizations, with the potential to improve and humanize care, recognizing the role of individuals, providers, families and the wider context in advancing care [55, 56].

## 5 Strengths and Limitations

There is a shortage of qualitative studies exploring the perspectives and experiences of individuals living with SMI and family members regarding the delivery of integrated physical and mental healthcare for this population. This study, guided by a Lived Experience Advisory Council, adds to the growing literature, offering insights and experiences from the Canadian context. As the majority of our participants were recruited in Ontario, Canada's most populous province, the generalizability of our findings to other areas in Canada, particularly rural and remote areas, which face unique challenges in accessing health resources, may be somewhat limited. Furthermore, the study focused on individuals with the most severe psychiatric disabilities, such as those served by ACT teams or treated in long‐term inpatient psychiatric units. As such, findings may not reflect the experiences and preferences of those less impacted by mental illness, who may face fewer barriers to accessing primary care services in the community. Further, in this study, we did not examine how the chronicity and symptom burden of comorbid physical health conditions might influence the experience and perceptions of access to physical healthcare, which should be the focus of future research. Despite these limitations, the study establishes an important foundation for future research, which could expand to include a broader range of key stakeholder groups, including providers, and participants from underrepresented areas and equity‐seeking groups, that may have unique needs and preferences.

## 6 Conclusion

Health providers, patients, family members and policymakers should work collaboratively to improve access to physical healthcare for adult living with SMI. Findings from this study can inform efforts to improve access to physical healthcare and whole‐person health in healthcare settings serving individuals with serious mental illness in Canada and other settings facing similar challenges.

## Author Contributions

**Munazzah Ambreen:** writing – original draft, writing – review and editing, formal analysis, data curation, software, investigation. **Christopher Canning:** conceptualization, funding acquisition, writing – review and editing, formal analysis, investigation. **Brian Lo:** conceptualization, funding acquisition, writing – review and editing, investigation. **Sri Mahavir Agarwal:** writing – review and editing. **David Castle:** writing – review and editing. **Barna Konkolÿ‐Thege:** writing – review and editing. **Frank Sirotich:** conceptualization, funding acquisition, writing – review and editing. **Sanjeev Sockalingam:** writing – review and editing. **Tania Tajirian:** writing – review and editing. **Philip G. Tibbo:** writing – review and editing, formal analysis. **Mary Rose Kesteren:** writing – review and editing, formal analysis. **Caroline Walker:** writing – review and editing, formal analysis. **Vicky Stergiopoulos:** conceptualization, funding acquisition, writing – original draft, methodology, validation, visualization, formal analysis, project administration, supervision, resources, investigation.

## Ethics Statement

Research Ethics Boards (REB) approval was obtained from CAMH (2023/057), the University of Toronto (00045386), Ontario Shores Centre for Mental Health Sciences (JREB# 23‐022‐R) and Dalhousie University (REB# 1030041).

## Consent

All participants provided written informed consent. A data‐sharing agreement was sought and fully executed between CAMH and Dalhousie University prior to before sharing data between these sites.

## Conflicts of Interest

Dr. Sri Mahavir Agarwal has received honoraria and speaker fees from HLS Therapeutics and Boehringer Ingelheim, Canada. All authors declare that the research was conducted in the absence of any commercial or financial relationships that could be construed as a potential conflict of interest.

## Supporting information

Supporting information.

## Data Availability

The datasets used and/or analysed are available from the corresponding author upon reasonable request with the approval of the institutional Research Ethics Board.
