# Supplementary material for: Strengthening the Delivery of Physical Healthcare for Adults Living With Serious Mental Illness – A Qualitative Description of Patient and Family Member Perspectives
Source: Health Expect. 2025 Mar 25;28(2):e70224. doi: 10.1111/hex.70224 (PMC11934217; doi:10.1111/hex.70224)
Supplement: Supplementary file 1 — Supporting information. [file HEX-28-e70224-s001.docx]

**Supplementary Appendix-1**

Interview Guide:

People with Serious Mental Illness (SMI)

# Part A: Lived Experience

In this first part of the discussion, the focus is on participants' lived experiences of accessing physical health monitoring within specialized mental health services, either for themselves or the person they cared for; and their thoughts on what ideal integrated care looks like. Try to probe for contextual detail; listen closely and aim to understand people and events in detail and the variety of circumstances under which experiences took place. Spend time up front, about 5-10 minutes, establishing a relationship and building rapport with the participants.

The questions below are a guide and should be adapted throughout the discussion; try to be flexible and responsive. For example, only ask questions the participant hasn’t yet addressed.

**Discussion questions are in bold**

“Thanks again for agreeing to do this interview. This study aims to provide feasible, acceptable, safe, effective, and patient centered policy options to support the delivery of integrated physical and mental health care in mental health settings in Canada and decrease health disparities for individuals with serious mental illness (SMI). Focus group discussions and interviews are being conducted with patients, carers, clinicians, policy makers and health administrators to understand different populations’ needs, perspectives and experiences towards integrating physical health care in mental health settings for people with SMI. The goal today is to understand your personal experiences of accessing physical health monitoring within specialized mental health services and your thoughts on what ideal integrated physical and mental health care might look like. In addition, we will discuss any assistance or care you have received with respect to accessing timely and appropriate physical health monitoring, and your opinions about what is helpful and not helpful.

In this first part of the discussion, I’d like to talk about your personal experiences.”

- 1. **The first question I’d like to ask is what made you interested to participate in this study of integrated care for people with serious mental illness?**
  2. **How would you describe your experience with accessing mental health care and physical health care so far?**
  3. **How do you perceive the level of concern and involvement your mental health providers have for your physical health?**

Prompts:

- - - Does your mental health providers know of any physical discomfort you may be experiencing?
    - In your experience, how often do mental health providers ask youabout physical health concerns during appointments or visits?
  1. **How have you and your mental health providers addressed medication side effects in your treatment plan, and what strategies have been most helpful in managing these side effects?**
  2. **Are you regularly screened for common health conditions such as diabetes, heart disease or cancer?**

(If yes) **Is there someone who usually reminds you to do so?**

(If no) **What prevents you from obtaining it?**

- 1. **How well have you been supported in navigating between multiple services?** Prompts:
     - Can you describe your experience of navigating between different services or providers for your mental health and related needs, and how easy or difficult this process has been for you?
     - How well do you feel that different providers or services have communicated with each other about your needs and care, and have there been any challenges or gaps in this communication?
  2. **Have you ever encountered any frustrating or challenging experiences when seeking assistance with your physical health?**

Prompts:

-If participant has trouble thinking of times they experience difficulties, or to supplement the discussion, ask:

-Seeking help at the Emergency Room

-Seeking help as an outpatient or through your family physician?

-Seeking or receiving help as an inpatient

- 1. **Have the mental health and physical health care providers been communicating effectively with one another to develop a comprehensive treatment plan for you? Can you describe the impact this had on your care experience?**
  2. **Can you describe any positive experiences you have had with coordinated physical and mental health care services?**
  3. **What kind of physical health supports or monitoring would you like to receive from your mental health providers? What are your reasons for choosing them?**
  4. **What types of support or resources do you think would be helpful in making and sustaining positive changes in your health risk factors or lifestyle factors, such as diet, exercise, or stress management?**
  5. **Have you received any support or resources from outside of your mental health and physical health care providers to help manage your overall health and well-being?** (If yes)
     - **Are there any specific organizations, community programs, or support groups that have been particularly helpful in supporting your overall health and well-being?**
     - **How do you think mental health and physical health care providers could work more effectively with other organizations and resources to better support patients with serious mental illness?**

# Part B: Structured Inquiry – Perspectives on Context

Following from participants’ lived experiences, the second half of the discussion shifts the focus toward their perspectives on the specific needs of different populations such as older adults, women, racialized participants, etc.

- 1. **What are your thoughts on the additional needs of some groups, such as women, older adults, or people from diverse cultural backgrounds or sexual and gender identities? Can you think of any additional needs?**

**a) Is there anything we could add or modify delivering integrated care to make it *more***

**helpful to these groups?**

# Part C: Wrap-up and conclusion

When you have the information you need and are ready to wrap up, you can note that your time together is ending, *briefly* summarize what you’ve heard, thank for their participation, and then consider one or more of the following wrap-up questions:

- **Is there anything else you would like to add?**
- **Is there anything else I should know?**
- **How did the discussion feel for you?**
- **How are you feeling now?**
- When the conversation is complete, turn off the audio-recorder

Focus Group Guide:

Family Members of People with SMI

# Part A: Lived Experience

In this first part of the discussion, the focus is on participants' lived experiences of accessing physical health monitoring within specialized mental health services for the person they cared for; and their thoughts on what ideal integrated care looks like. Try to probe for contextual detail; listen closely and aim to understand people and events in detail and the variety of circumstances under which experiences took place. Spend time up front, about 5-10 minutes, establishing a relationship and building rapport with the participants.

The questions below are a guide and should be adapted throughout the discussion; try to be flexible and responsive. For example, only ask questions the participant hasn’t yet addressed.

**Discussion questions are in bold**

“Thanks again for agreeing to do this focus group discussion. This study aims to provide feasible, acceptable, safe, effective, and patient centered policy options to support the delivery of integrated physical and mental health care in mental health settings in Canada and decrease health disparities for individuals with serious mental illness (SMI). Focus group discussions and discussions are being conducted with patients, carers, clinicians, policy makers and health administrators to understand different populations’ needs, perspectives and experiences towards integrating physical health care in mental health settings for people with SMI. The goal today is to gain insights into your personal experiences of assisting the person you care for in accessing physical health monitoring within specialized mental health settings. We are also interested in hearing your thoughts on what an ideal integrated physical and mental health care system would encompass. Additionally, we will discuss any assistance you have received regarding timely and suitable physical health monitoring for the individual you care for, and we value your opinions on what has been helpful and what has not.

In this first part of the discussion, I’d like to talk about your personal experiences.”

**1. The first question I’d like to ask is what made you interested to participate in this study of integrated care for people with serious mental illness?**

1. **How would you describe your experience with helping the individual you take care of in accessing mental health care and physical health care so far?**
2. **How would you describe the level of concern and involvement your mental health providers show towards the physical health of the person you care for?**

Prompts:

- - 1. Do your mental health providers inquire about any physical discomfort that the person you care for may be experiencing?
    2. Based on your experience, how frequently do mental health providers ask about physical health concerns during appointments or visits with you or the person you care for?

1. **How have the mental health providers addressed medication side effects in the treatment plan for the person you care for, and which strategies have proven most helpful in managing these side effects?**
2. **Is the person you care for regularly screened for common health conditions such as diabetes, heart disease, or cancer?**

(If yes) **Is there someone who usually reminds them to do so?**

(If no) **What prevents them from obtaining it?**

1. **How well have you been supported in navigating between multiple services for the mental health and related needs of the person you care for?**

Prompts:

- - 1. Can you describe your experience of navigating between different services or providers for the mental health and related needs, and how easy or difficult this process has been?
    2. How well do you feel that different providers or services have communicated with each other regarding their needs and care, and have there been any challenges or gaps in this communication?

1. **Have you ever encountered any frustrating or challenging experiences when seeking assistance with the physical health of the person you care for?**

Prompts:

-If participant has trouble thinking of times they experience difficulties, or to supplement the discussion, ask:

-Seeking help at the Emergency Room

-Seeking help as an outpatient or through your family physician?

-Seeking or receiving help as an inpatient

1. **Have the mental health and physical health care providers been communicating effectively with one another to develop a comprehensive treatment plan for the person you care for? Can you describe the impact this had on their care experience?**
2. **Can you describe any positive experiences you have had with coordinated physical and mental health care services for the person you care for?**
3. **What kind of physical health support or monitoring would you like the mental health providers to provide for the person you care for? What are your reasons for choosing these types of support?**
4. **Have you received any support or resources from outside of your mental health and physical health care providers to help manage the overall health and well-being of the person you care for?** (If yes)
   - 1. **Are there any specific organizations, community programs, or support groups that have been particularly helpful in supporting their overall health and well-being?**
     2. **How do you think mental health and physical health care providers could work more effectively with other organizations and resources to better support patients with serious mental illness?**

# Part B: Structured Inquiry – Perspectives on Context

Following from participants’ lived experiences, the second half of the discussion shifts the focus toward their perspectives on the specific needs of different populations such as older adults, women, racialized participants, etc.

1. **What are your thoughts on the additional needs of some groups, such as women, older adults, or people from diverse cultural backgrounds or sexual and gender identities? Can you think of any additional needs?**

**a) Is there anything we could add or modify delivering integrated care to make it *more***

**helpful to these groups?**

# Part C: Wrap-up and conclusion of the discussion

When you have the information you need and are ready to wrap up, you can note that your time together is ending, *briefly* summarize what you’ve heard, thank the participants for their participation, and then consider one or more of the following wrap-up questions:

- **Is there anything else you would like to add?**
- **Is there anything else I should know?**
- **How did the discussion feel for you?**
- **How are you feeling now?**

The following guidelines will help to ensure a smooth, productive and respectful discussion:

- When the conversation is complete, turn off the audio-recorder
